# The Role of Education in Emotional Intelligence to Perceive, Understand and Regulate Emotions: A Quasi-Experimental Study

**DOI:** 10.3390/healthcare13131542

**Published:** 2025-06-27

**Authors:** Silvia Évora-Lebrero, Marta Bustos-Sepúlveda, Lluvia Bustos-Sepúlveda, Antonio Segura-Fragoso, Victor Florez-Garcia, Kaboni Whitney Gondwe, Esmeralda Santacruz-Salas

**Affiliations:** 1 Southeast University Hospital, Arganda del Rey, 28500 Madrid, Spain; 2 Assistance to People—ASISPA Foundation, 28027 Madrid, Spain; 3 Arcosur Integrated Public School in Zaragoza, Education Technician of the Government of Aragon, 50022 Zaragoza, Spain; 4 Institute of Health Sciences of Castilla-La Mancha, Talavera de la Reina, 45600 Toledo, Spain; 5 Department of Public Health, Universidad del Norte, Barranquilla 080003, Colombia; 6 Division of Epidemiology & Biostatistics, University of Illinois Chicago, Chicago, IL 60607, USA; 7 Child, Family and Population Health Nursing, School of Nursing, University of Washington Seattle, Seattle, WA 98195, USA; 8 Faculty of Physiotherapy and Nursing of Toledo, University of Castilla-La Mancha, 45004 Toledo, Spain

**Keywords:** childbirth, emotion, emotional intelligence, mHealth, postnatal care, pregnancy

## Abstract

**Background:** Emotional intelligence is the ability to make decisions and adapt to life changes. This capacity may be conditioned by emotional states. Evidence shows that postpartum women experience these changes, which affect an estimated 80% of postpartum women and their parenting management. The purpose of this study was to analyze the impact of interventions based on reinforcing emotional intelligence in pregnant and postpartum women and their relationship with the different sociodemographic and clinical characteristics of the mother and newborn. **Methods:** We conducted a quasi-experimental experiment between August 2016 and December 2018. We recruited a total of 69 pregnant women (35 women in the intervention group and 34 women in the control group). The pregnant women in the intervention group were exposed to hybrid interventions through a mobile health application and in-person interventions as part of a training and emotional management program. The Trait-Meta Mood Scale (TMMS-24) evaluation questionnaire was administered to measure emotional intelligence across its three dimensions. **Results:** The results showed important clinical significance, although not statistical significance in all TMMS domains. Postpartum scores for clarity (mean: 29.20; SD 6.36 vs. 24.91; SD 6.67; *p* > 0.05), repair (mean: 28.43; SD 5.58; vs. 24.62; SD 7.04; *p* > 0.05), and attention (mean: 26.03; SD 6.08 vs. 23.29; SD 5.14; *p* > 0.05) were higher in the intervention group compared to the control group. The duration of gestation notably influenced “Clarity”, while work situations and parental support affected “emotional repair”. **Conclusions:** Educational training increased emotional competencies and equipped women with the emotional mechanisms needed to adopt new adaptation strategies. Training in emotional self-management during pregnancy has a greater impact on the “Clarity” of emotions.

## 1. Introduction

Emotional intelligence (EI) is a complex concept encompassing aspects not only related to intelligence but also quotient or academic abilities [1] and the ability to use reasoning and abstract thinking in decision making and problem solving, which is conditioned by emotional states that reflect external factors and internal behaviors [2]. This also considers individual’s social and practical characteristics or skills such as self-control, enthusiasm and perseverance, and the ability to motivate oneself [3]. Evidence shows that EI is associated with adaptation to the environment and mood regulation. Therefore, EI can influence interpersonal relationships, the perception of the achievement of objectives and goals, greater emotional and stress control and regulation and less depressive symptoms [4,5,6].

The perinatal period is critical for women as it involves multiple changes: physiological, hormonal, emotional, behavioral and cognitive. As women adapt to pregnancy and childbirth, they experience fears, doubts or insecurities regarding the new situation that awaits them. Some women adapt well to these changes, while others may experience stress and anxiety [7]. Different studies have demonstrated the relationship between certain emotional or psychological characteristics of a woman and their impact on the evolution of pregnancy, the postpartum period and the baby [8,9]. For example, perinatal stress, depression, low self-esteem or anxiety are negatively correlated with maternal [10,11] and infant outcomes [12]. Specifically, prenatal anxiety is associated with adverse pregnancy and infant outcomes [13,14,15]. There may also be long-term consequences for children’s mental health [16,17]. One study showed that 61% of the children of mothers with depression presented experienced anxiety disorders and oppositional defiant behaviors compared to 15% in cases with mothers without depression [18]. The relationship between anxiety and depression is not new. Several studies have shown that excess cortisol secretion increases vulnerability to anxiety and is positively related to stress [19].

Postpartum mood disorders can affect up to 80% of women and lead to other health problems [20,21]. These may present as symptoms of “postpartum blues” characterized by sadness and apathy [22]. But they can also manifest themselves in severe cases, where the intensity of sadness or its duration persists, even leading to suicidal thoughts. The presence of a major depressive disorder at this stage can interfere with motherhood and parenting, cause a loss of control and feelings of an inability to be a mother and difficulty breastfeeding [23].

The causes of mood disorders, peripartum depression and predisposing factors are currently unknown. However, several studies confirm that there is a contribution from genetic, environmental, nutritional and psychosocial factors [24,25] and that the strongest predictor is depression during pregnancy [26]. Whether postpartum depression is a cause or a consequence, the repercussions affect the environment of mothers and their families.

The importance of early detection will increase the possibilities of intervention and treatment that can prevent harmful consequences for both the mother and the newborn. Therefore, it is essential that health professionals consider examining the emotional state of the mother during their pregnancy and postpartum check-ups to detect any depressive symptoms and implement appropriate interventions. It has been reported that more than 50% of mothers do not have the help of friends or family. Furthermore, it is difficult for them to express their emotional problems to the health professionals who attend to their obstetric process [27]. Although research on emotional intelligence in perinatal care has grown, including recent systematic reviews [28], more intervention studies are needed to assess its practical impact on maternal well-being and adjustment during the transition to motherhood. Most studies focus on child development. This helps consolidate the intensive motherhood model and increases social and internal pressure on mothers [14,29]. Motherhood must be valued and studied to help those mothers who suffer in this process to recover their value as women, regardless of the rewarding processes that the mother–child relationship may include [30]. Thus, we carried out an intervention based on the reinforcement of EI in pregnant and postpartum women, to evaluate whether support in the management of stressful situations in a personalized way before childbirth and the first months after childbirth could prevent or improve the symptoms of the “postpartum blues” and meet a woman’s emotional needs.

We start from the premise that there is a positive association between the improvement of emotional intelligence skills, emotional control, and regulation, which could help in the management of depressive symptoms after postpartum. This study adopts the Mayer and Salovey ability model of emotional intelligence, which conceptualizes EI as a set of cognitive–emotional abilities including emotional perception, understanding, and regulation, core components reflected in the Trait-Meta Mood Scale (TMMS-24) dimensions [31]. Therefore, the purpose of this study was to analyze the effectiveness of interventions based on reinforcing EI in pregnant and postpartum women through a new educational project for emotional management, called “Happy in My Motherhood”. We hypothesized that women who received the perinatal EI reinforcement intervention would report higher EI compared to women who received the current standard of care. As secondary objectives, we plan to assess the relationship between the impact of the program and the different sociodemographic and clinical characteristics of the mother and newborn.

## 2. Methods

### 2.1. Research Design

We carried out a quasi-experimental study titled “Happy in my Motherhood.” Pregnant women were included between August 2016 and December 2018. All participants provided informed consent, including for the use of the mobile application developed for this study. Ethical approval was obtained from the Ethics and Research Committee of the Hospital Universitario Sureste de Madrid (Code: 13/2015).

### 2.2. Intervention

The intervention involved activities from the growth and improvement of emotional skills program, “Happy in my Motherhood”, which were based on two face-to-face educational sessions in the hospital for 90 min, between weeks 28 and 34 of gestation. In them, training was offered on the different types of emotions, their purpose, management and control tools as basic meditation guidelines. At the end of each session, they were also provided, via the app, with supportive written material related to the session. Likewise, the IG received weekly written support material through the application during the first eight weeks postpartum. This material addressed the most common types of emotions experienced during the postpartum period and offered physical activity exercises to do at home. Finally, these women were additionally provided with text messages with emotional support, as well as an email address through which they could clarify doubts, request additional information and/or share their emotional experiences.

### 2.3. Recruitment

**Figure 1:**
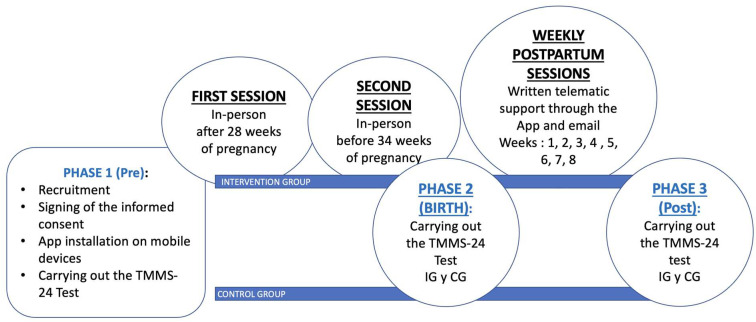
Diagram of activities and interventions in the “Happy in My Motherhood” program.

The recruitment of women was carried out during the fortnightly information sessions held for pregnant women in our hospital facilities during the period 2016–2018. They were informed of the study and their participation was requested. Those who offered their willingness to participate signed the informed consent form and attended the preparation for childbirth session with the midwives. They were assigned “by convenience” to our intervention group (IG) or control group (CG). On the day of recruitment, all women installed a mobile application (app) called “Happy in my Maternity” under the supervision of the researcher, which was created and designed specifically for the study. All study follow-up information was collected through this application. The information offered to all mothers through the mobile application included a welcome message, contact information, sociodemographic and clinical variables, and access to the TMM-24 questionnaire. However, the GI, through the mobile application, had access to informative and training content regarding the CG. The GI mothers received additional training material in the first two face-to-face prenatal sessions and new information during the postnatal intervention period, between weeks 1 and 8. (Figure 1).

### 2.4. Population

Women were eligible to participate if they met the following criteria: (i) aged 18 years and older, (ii) between 28 and 38 weeks pregnant, (iii) pregnant women undergoing follow-up and control at the participating hospital, and (iv) had the ability to provide consent participate in the study. Exclusion criteria included high-risk pregnancies; women with psychiatric disorders before pregnancy, such as anxiety and affective or disruptive behavior disorders; and having been admitted to a neonatal intensive care units after delivery.

A total of 147 women gave their consent to participate in the study. We excluded 78 women due to a lack of response to the questionnaires: 31 women in the first visit, 15 women in the second and 23 in the last visit. Additionally, nine women were excluded because their newborns had health problems after delivery. Finally, 35 women were included in the intervention group and 34 in the control group.

### 2.5. Sample Size Calculation

We estimated the sample size using the difference between the mean scores on the Trait-Meta Mood Scale (TMMS 24) in the independent groups. Considering an initial mean of 70 points with an SD of 11 points, an expected difference after the intervention of 6.6 points (Cohen’s D effect size of 0.6 = large effect) with an error probability alpha = 0.05 and statistical power (1-probability of beta error) = 0.8, the minimum sample size was 72 women (36 per group).

## 3. Measures

Data was collected at three timepoints: Prenatal (Phase 1, during the third trimester of pregnancy and prior to delivery), Birth (Phase 2-during the first 24 h after delivery) and Postnatal (Phase 3, 8 weeks after delivery)

### 3.1. Maternal Characteristics Variables

Maternal demographics and characteristics were collected using self-report questionnaires on maternal age, education, marital status, nationality, mode of delivery, type of anesthesia, parity, conception type, breastfeeding status, gestational age, support from parents, support from partner, support from family members, support from external sources, and infant sex.

### 3.2. Emotional Intelligence

We measured EI using the Trait-Meta Mood questionnaire Scale (TMMS-24), which measures the self-regulation and emotional skills of mothers. The TMMS-24 questionnaire is based on the previous version developed by Salovey et al., which was adapted and translated into Spanish [31]. The TMMS-24 test is a validated instrument to measure general emotional intelligence and trait emotional intelligence [32,33]. It consists of 24 items, which will be answered on a 5-point Likert scale. It can be used to identify skills to interpret and manage emotions. This questionnaire evaluates three components, dimensions or domains of Emotional Intelligence: (A) Emotional Perception or Attention, which refers to our ability to recognize our feelings and become aware of our emotions; (b) Understanding or Clarity in the Emotions of one’s own feelings. This is related to the ability to understand and recognize different emotional states well, understand the changes and properly integrate them into thinking; and (c) Emotional Regulation or Emotional Repair, which is related to the ability to regulate and control positive and negative emotions. The TMMS-24 was selected due to its strong psychometric properties, cultural adaptation, and validation in Spanish-speaking populations. Its brevity and ease of administration also make it particularly suitable for use with pregnant and postpartum women, facilitating adherence and minimizing participant burden.

### 3.3. Statistical Analysis

A descriptive analysis of sociodemographic and pregnancy variables was carried out using absolute frequencies and percentages for each category, comparing the intervention and control groups using the chi-square test. Quantitative variables such as age, Emotional Attention, Emotional Clarity and Emotional Repair were described using the mean and standard deviation (SD).

We used repeated measures ANOVA models with group (intervention/control) as the between-subjects variable and time (pre-intervention, 24 h and post-intervention) as the within-subjects variable. The effect size was determined using partial eta squared. To describe the effect of the intervention, the differences in the questionnaire scores at the three time points were calculated for each of the groups independently. The times correspond to Phase 1 (before delivery), Phase 2 (24 h after delivery) and Phase 3 (after completion of the intervention sessions, 8 weeks after delivery) for the intervention group and the control group. The net differences between the results offered at the three moments, Phase 1 (prenatal), Phase 2 (birth) and Phase 3 (postnatal), between the intervention group and the control group were also analyzed. For each of the dependent variables (Emotional Attention, Emotional Clarity and Emotional Repair), a general linear model of repeated measures was fitted with a within-subjects factor (measurement 1 = pre; measure 2 = 24–48 h; and measure 3 = post) and a between-subjects factor (experimental control group). The overall effect of the intervention in the control group was calculated using eta squared and its *p* value. In addition, the chi-square test was used to compare the categories of each sociodemographic variable, the percentages of women who presented scores above the respective median in Attention, Clarity, Repair and Total. The level of statistical significance was established at *p* < 0.05. The statistical analysis was performed using IBM SPSS v24. This study was reviewed and approved by the Research Ethics Committee of the Southeast University Hospital (HUS) (Code: #13/2015).

## 4. Results

### 4.1. Description of the Sample

Table 1 shows the sociodemographic and clinical characteristics of the women studied in relation to the emotional education program to improve emotional intelligence skills. The mean age was 33.1 ± 4.4 years; the majority had completed advanced university studies (43.5%). Furthermore, 72.5% of births were normal and most women required epidural anesthesia (81.2%).

### 4.2. Effect of the Intervention

Table 2 presents the mean Emotional Attention, Clarity of Feelings, and Emotional Repair for both the intervention and control groups. While the intervention group showed higher mean values across all three domains in the postnatal phase, including greater Emotional Repair and Clarity, these between-group differences were not statistically significant.

Table 3 shows the differences that the women presented independently, in both the control and intervention groups, comparing the results of the TMMS-24 Test in Phase 1 (Prenatal), Phase 2 (24 h Postnatal Period), and Phase 3 (Postnatal Period) at the end of the 8-week postpartum period. Significant mean differences were seen in Emotional Repair and Clarity of Feelings at all time points. While mean differences in Emotional Attention were not statistically significant, the results are still clinically significant as the intervention group still had higher scores compared to the control group.

Table 4 shows the difference attributable to the intervention, as well as the magnitude of the effect (partial *eta* squared) and the statistical significance of the group by temporal interaction. Our findings showed that although postpartum scores for Clarity (mean: 29.20 vs. 24.91; *p* > 0.05) and Repair (mean: 28.43 vs. 24.62; *p* > 0.05) were higher in the intervention group compared to the control group, these differences were not statistically significant in the cross-sectional comparison in the postnatal phase. However, repeated measures analysis (Table 4) showed a significant time-by-group interaction effect in the Clarity dimension (partial *eta* squared: 0.15) and statistical significance (*p* = 0.00), indicating that the intervention had a meaningful longitudinal impact. A small and non-significant effect was seen for Emotional Attention, a medium effect that almost reached statistical significance was seen for Emotional Repair and the intervention had a large and statistically significant effect of Clarity of Emotions. Nonetheless, the consistent trend toward higher scores in the intervention group suggests a potentially meaningful clinical effect, further supported by the longitudinal analyses presented in Table 4.

**Figure 2:**
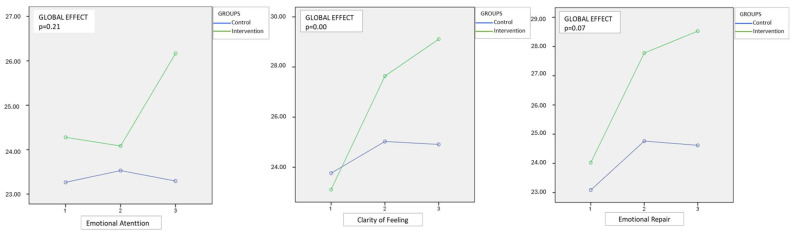
Comparative results for Emotional Attention, Clarity of Feelings and Emotional Repair in the TMMS-24 questionnaire in the follow-up period.

Figure 2 graphically shows the separate results for each of the Emotional Attention, Emotional Clarity and Emotional Repair dimensions, respectively, of the TMM-24 test in the three time periods. Regarding Emotional Care, the women who received the interventions and therefore the training with the “Happy in My Maternity” program improved in this domain, especially in the period between childbirth (24 h) and the end of the program. (Post). The women in the CG had practically no changes in their results. Therefore, there is an effect of the intervention, which is statistically significant between the 24 h and Post periods.

The statistical analysis carried out to evaluate the relationship between the sociodemographic characteristics collected and the results of the TSMM-24 questionnaire in each dimension did not show statistical significance except in the dimensions Clarity (*p* = 0.02) and Emotional Attention (*p* = 0.08). For Emotional Clarity, women aged 30 or older scored higher than younger women (n = 30, 58.8% vs. n = 5, 27.8%). In Emotional Care, those who had complications during childbirth (n = 19, 63.3%) presented higher scores (n = 26, 42.1%) compared to those who did not have them. There were no statistically significant results in relation to the sociodemographic variables and Emotional Repair.

## 5. Discussion

This study assessed the implementation of an educational program in women in the perinatal period to improve their abilities and provide tools for emotional management and control. The results of this study highlight the importance of the adequate development of protective factors such as emotional intelligence during vulnerable stages and biopsychosocial changes such as the postnatal period. It is important to reflect on the effects and impact of emotional self-regulation practices and support for women at this stage of their lives. Also, enhancing emotional intelligence during the perinatal period may not only benefit individual well-being but also fosters prosocial behaviors, strengthening social support and maternal–infant bonding [34].

### 5.1. Dimensions or Domains of the Emotional Intelligence Questionnaire (TMMS 24)

The three components of emotional intelligence showed significant increases in their scores in the IG women compared to the CG, with Clarity of Emotions also being statistically significant. The magnitude of the effect of the intervention measured by the *partial eta square statistic* shows a significant effect of the program (Eta^2^ 0.15) in the women who received the training in the Emotional Clarity dimension and a medium effect in Emotional Repair (Eta^2^ 0.05). Although women who received training and help from the program improved their Emotional Care scores much more than those who did not, the effect was small (Eta^2^ 0.03). This result is consistent with existing evidence from studies that utilized the questionnaire [32,35,36]. Emotional Attention evaluates how much a person pays attention to their emotional states and how much awareness they have of their feelings. It is a measure of emotional self-awareness; that is, the ability to identify and recognize one’s own emotions clearly and accurately. Therefore, providing support in these areas to women in the perinatal period strengthens and improves their ability to notice and pay attention to their own emotions. Being aware of your own emotions is the first step to being able to manage them effectively. If a person pays little attention to their emotions, they may have difficulties understanding and regulating their emotional states, which can affect their psychological well-being and interpersonal relationships. However, high scores in Emotional Attention can lead to excessive monitoring of emotions and sensations, which can also lead to worries and disturbances. However, the opposite is true with Emotional Clarity and Repair. In this case, high scores in these dimensions lead to better control and management of intrapersonal emotions [35,36].

### 5.2. Relationship with Sociodemographic Characteristics of the Sample

Some studies have established associations between specific sociodemographic factors and the prevalence of psychopathological or emotional problems. For example, some authors have previously explored a positive link between emotional intelligence and various practices such as breastfeeding and labor [36,37]. However, we have not found any literature that analyzes programs to improve emotional intelligence and its correlation with sociodemographic variables. The results of this work can serve as a basis for future research since the findings show certain associations between some sociodemographic and clinical characteristics and an improvement in the acquisition of emotional intelligence skills after an emotional education program during pregnancy and the postpartum period. Our research showed that women aged 30 years or older (*p* 0.03), with a higher educational level (*p* 0.03) and longer gestation periods (*p* 0.05), show superior results in terms of their emotional capacities. According to some authors, an emotional adaptive process involves perception, evaluation and regulation [38]. These phases correspond to the dimensions of the TMMS-24 Emotional Intelligence questionnaire: Emotional Attention (perceive and pay attention to emotions), Emotional Clarity (understand and value emotions) and Emotional Repair (regulate and manage emotions). In this sense and according to the total results of the questionnaire, we could affirm that time, pregnancy and age, as well as the highest educational level, have a positive effect on the control of emotional states.

Furthermore, when the sociodemographic variables were analyzed in contrast to each of the dimensions of the questionnaire, independently, women over 30 years of age also significantly improved their scores in the Emotional Clarity items (*p* = 0.02).

### 5.3. Relationship Between the Different Phases of the Study

In this pioneering program of emotional education in the perinatal period, we have observed clear differences between the moments of data collection in the IG and the CG. In the dimension of Attention or emotional perception, the results show that the women who received the program interventions improved their scores in the questionnaire. Furthermore, these results were statistically significant (*p* = 0.02) between the last examination (Post) and birth. Women in the CG who did not receive the training showed virtually no change in their scores during the follow-up period, while those included in the program improved by almost two points. Therefore, pregnant and postpartum mothers who had not benefited from the “Happy in My Motherhood” program paid less attention to their emotions, and therefore did not identify their emotional states as clearly. The program helped the women focus on their emotions. Detecting emotions and identifying them is the basis for being able to take control of them later.

In the dimension Clarity of Emotions or understanding, in the latest results regarding the answers to the first test before childbirth, the CG women barely showed an improvement in the mean scores, with a difference of 1.15 between the last test (Post) and the first (Pre). However, women included in the program increased by six points during the same period. This means that the face-to-face sessions, with continuity in the emotional support provided in the “Happy in My Motherhood” program, are highly effective (*p* = 0.00) for clarifying, discerning and understanding emotions. This dimension of the test offers better results, being statistically significant in the analysis of any of the comparisons carried out in the IG. Research has shown that having Clarity in Emotions can act preventively in the face of a stressor [38]. People with higher scores in this dimension were less fatigued and sad after being exposed to the stressor. This could be important for women at this stage of their lives, including pregnancy and postpartum.

In the dimension of Repair/Emotional Regulation, as in the dimension of Clarity of Emotions/Emotional Repair, there was a greater improvement in the experimental cases than in the women in the CG. The results show an increase of 4.5 points on the TMMS-24 scale for women who received training and support in emotional intelligence compared to 1.53 for the others. This leads us to conclude that the “Happy in My Motherhood” program increases the ability of postnatal mothers to more effectively regulate their emotional states. Other authors have shown evidence that those with higher scores for Emotional Repair showed lower scores for depression and anger [39,40]. Therefore, this condition would help the mother maintain greater control of her emotional state and counteract or prevent depressive symptoms [36].

It is also worth mentioning that, for the experimental group, the best scores were obtained in the Clarity of Emotions and Emotional Repair dimensions, and were inversely proportional to what the women in the CG showed. These women showed a decrease in their scores in the three dimensions of the test in Phase 3 (post) compared to the results related to Phase 2 (first 24 h after delivery). This shows that mothers who lack emotional control and do not receive emotional support can become overwhelmed by the influx of events after childbirth. Their scores on the TMM-24 test dropped in these first weeks and their Emotional Attention, Understanding, and Repair capacities may have been impaired. As other authors have stated, it is important to prevent emotional variability.

While the intervention was associated with improvements in emotional intelligence scores, these results should be interpreted with caution given the quasi-experimental design. Although the findings do not provide direct evidence for preventive effects on postpartum depression, they suggest a potential benefit for emotional self-regulation during the perinatal period. Helping women develop adaptive coping tools through emotional self-management may contribute to a more rewarding and balanced experience of motherhood for both them and their children [41]. Given the observed improvements in emotional competencies, particularly during pregnancy, it may be beneficial to consider the integration of emotional training programs into prenatal care. Hospital-based units or midwife-led prenatal classes could serve as suitable platforms for implementing such interventions, ultimately aiming to enhance maternal well-being and quality of life during the postnatal period.

## 6. Limitations and Strengths of the Study

Some of the most important limitations of this study include the small sample size and the use of a single measurement instrument, the TMMS-24 questionnaire. Additionally, the use of a convenience-based sample may have introduced selection bias, limiting the generalizability of the findings. The quasi-experimental design also restricts causal interpretations.

A particularly relevant limitation is the high attrition rate (53% of initially enrolled participants), which may have introduced attrition bias. No sensitivity analysis was conducted to assess differences between completers and dropouts, which limits the ability to evaluate the robustness of the results. Moreover, the sample size calculation was based on a large, expected effect size (Cohen’s d = 0.6), which may be optimistic, as smaller effects are more typical in psychosocial interventions. Future research should consider more conservative estimates based on the existing literature.

To enrich the understanding of emotional intelligence development during the perinatal period, future studies should incorporate multivariate regression models to explore interactions between sociodemographic variables and outcomes. Including qualitative data could also provide valuable insight into individual experiences and emotional processes.

Despite these limitations, the strength of this study lies in its integrative approach to promoting emotional care for women during pregnancy and the postpartum period, aiming to minimize adverse outcomes and reduce healthcare burdens. Furthermore, the results align with previous evidence linking higher emotional intelligence to greater perceived social support and enhanced subjective well-being [32], underlining the potential of such interventions to strengthen both emotional and social resources in the perinatal context [37].

## 7. Conclusions

The “Happy in My Maternity” program provides emotional tools to future mothers to adapt to the changes in the first weeks of the postpartum period and shows effectiveness in the acquisition of skills for emotional perception, understanding of one’s own emotions and Emotional Regulation/management in those women who received intervention compared to those in the CG, being able to help them repair and/or prevent stressful situations or pathological emotional states more efficiently.

The development of skills through training and support in emotional intelligence for women in the last stage of pregnancy and the postpartum period increases emotional self-management.

Women aged 30 or older, with a university level of education or higher and with a pregnancy between 41 and 42 weeks of gestation, obtained higher scores in the instrument used for measuring emotional intelligence, the TMMS-24 questionnaire. The impact of the program in descending order would be first for Clarity in Emotions, followed by the capacity for Emotional Repair and finally for Emotional Attention.

## Figures and Tables

**Table 1:** The sociodemographic and clinical characteristics of the women studied in relation to the results obtained in the TMMS-24.

		Control Group	Group Intervention	TOTAL	*p* Value *
		n = 34 (%)	n = 35 (%)	n = 71 (%)	
**(Year old)**					
	<30	13 (72.2)	5 (27.8)	18 (26.1)	0.03
	> =30	22 (43.1)	29 (56.9)	51 (73.9)	
**Education level**					
	Secondary studies	3 (8.8)	1 (2.9)	4 (5.8)	0.03
	University Studies	8 (23.5)	7 (20)	15 (21.7)	
	Professional education	5 (14.7)	1 (2.9)	6 (8.7)	
	Advanced Professional Education	6 (17.6)	1 (2.9)	7 (10.1)	
	Postgrads studies	9 (26.5)	21 (60)	30 (43.5)	
	NC	3 (8.8)	4 (11.4)	7 (10.1)	
**Nationality**					
	Spain	31 (91.2)	31 (88.6)	62 (89.9)	0.41
	France	0 (0)	1 (2.9)	1 (1.4)	
	Italy	0 (0)	1 (2.9)	1 (1.4)	
	Romania	3 (8.8)	1 (2.9)	4 (5.8)	
	Venezuela	0 (0)	1 (2.9)	1 (1.4)	
**Type of delivery**					
	Cesarean section	7 (20.6)	7 (20)	14 (20.3)	0.88
	Eutocic vaginal delivery	24 (70.6)	26 (74.3)	50 (72.5)	
	Instrumental	3 (8.8)	2 (5.7)	5 (7.2)	
**Type of Anesthesia**					
	Spinal	1 (2.9)	1 (2.9)	1 (1.4)	0.66
	Epidural	26 (76.5)	30 (85.7)	56 (81.2)	
	General	2 (5.9)	1 (2.9)	3 (4.3)	
	None	4 (11.8)	3 (8.6)	1 (1.4)	
**Previous Children**					
	0	22 (64.7)	26 (74.3)	48 (69.6)	0.67
	1	11 (32.4)	8 (22.9)	19 (27.5)	
	2	1 (2.9)	1 (2.9)	2 (2.9)	
**Conception type**					
	In vitro	0 (0)	2 (5.7)	2 (2.9)	0.16
	Natural	34 (100)	33 (94.3)	67 (97.1)	
**EB in previous children**					
	No	3 (23.1)	4 (40)	7 (30.4)	0.38
	Yes	10 (76.9)	6 (60)	16 (69.6)	
**Gestation weeks**					
	36–40	29 (58)	21(42)	50 (72.46)	0.05
	41–42	6 (31.6)	13 (68.4)	19 (27.53)	
**Coexistence as a Couple**					
	NC	2 (5.9)	7 (19.4)	3 (4.3)	0.34
	No	1 (2.9)	0 (0)	1 (1.4)	
	Yes	31 (91.2)	34 (97.1)	65 (94.2)	
**Has help in parenting**					
	NC	2 (5.9)	3 (8.6)	5 (7.2)	0.11
	No	4 (11.8)	0 (0)	4 (5.8)	
	Yes	28 (82.4)	32 (91.4)	60 (87)	
**Couple Support**					
	NC	3 (8,8)	3 (8.6)	6 (8.7)	0.11
	No	4 (11.8)	0 (0)	4 (5.8)	
	Yes	27 (79.4)	32 (91.4)	59 (85.5)	
**Support for family members**					
	NC	3 (8,8)	4 (11.4)	7 (10.1)	0.65
	No	10 (29.4)	7 (20)	17 (24.6)	
	Yes	21 (61.8)	24 (68.6)	45 (65.2)	
**External Support**					
	NC	3 (8.8)	5 (14.3)	8 (11.6)	0.69
	No	22 (64.7)	23 (65.7)	45 (65.2)	
	Yes	9 (26.5)	7 (20)	16 (23.2)	
**Newborn sex**					
	Female	20 (58.8)	15 (42.9)	35 (50.7)	0.19
	Male	14 (41.2)	20 (57.1)	34 (49.3)	

* Chi-square test. NC = did not answer; EB in previous children = exclusive breastfeeding in previous children.

**Table 2:** Results fpr Emotional Attention, Clarity of Emotions and Emotional Repair in different phases of the study.

Questionnaire TMMS-24	Control Group		Intervention Group	
	n = 34 (Half of)	95% CI	n = 35 (Half of)	95% CI
Emotional Care–Pre	23.26 (5.09)	21.55; 24.98	24.26 (5.10)	22.57; 25.95
Emotional Care–Birth	23.53 (5.89)	21.55; 25.51	24.03 (4.82)	22.43; 25.62
Post-Emotional Care	23.29 (5.14)	21.57; 25.01	26.03 (6.08)	24.01; 28.04
Clarity of feelings–Pre	23.76 (5.35)	21.96; 25.53	23.29 (5.88)	21.34; 25.23
Clarity of feelings–Birth	23.03 (5.50)	23.18; 26.87	27.66 (6.437)	25.51; 29.80
Clarity of feelings–Post	24.91 (6.67)	22.67; 27.12	29.20 (6.36)	27.09; 31.31
Emotional Repair–Pre	23.09 (6.11)	21.04; 25.15	24.20 (4.56)	22.69; 25,713
Emotional Repair–Birth	24.76 (6.29)	22.65; 26.86	27.74 (5.89)	25.79; 29.70
Post-Emotional Repair	24.62 (7.04)	22.25; 26.93	28.43 (5.58)	26.58; 30.28

Pre = period before childbirth or third trimester of pregnancy; Birth = first 24 h after birth; Post = completion of the puerperium, first 8 weeks after childbirth. 95%CI = confidence interval; TMMS = Trait-Goal Mood Scale.

**Table 3:** Differences in mean scale scores in the emotional education program “Happy in my Motherhood”.

QuestionnaireTMMS-24	Control Group			Group Intervention		
	-Pre Birth	Post-Pre	Post-Birth	-Pre Birth	Post-Pre	Post-Birth
	Mean Difference (*p* Value)	Mean Difference (Value *p*)	Mean Difference (Value *p*)	Mean Difference (Value *p*)	Mean Difference (Value *p*)	Mean Difference (Value *p*)
Emotional Attention	0.26 (0.75)	0.03 (0.97)	−0.24 (0.71)	−0.23 (0.77)	1.77 (0.09)	2 (0.02)
Clarity of Feelings	1.26 (0.06)	1.15 (0.24)	−0.12 (0.89)	4.37 (<0.001)	5.91 (<0.001)	1.54 (0.03)
Emotional Repair	1.68 (0.03)	1.53 (0.11)	0.15 (0.84)	3.54 (<0.001)	4.23 (0.001)	0.69 (0.44)

Pre = period before childbirth or third trimester of pregnancy; Birth = first 24 h after birth; Post = Completion of the puerperium, first 8 weeks after childbirth; TMMS = Trait-Goal Mood Scale.

**Table 4:** Net difference in intervention and magnitude of the effect.

TMMS-24 Quiz	Differences Between Groups at Different Times			Magnitude of the Effect. Interaction Between Groups by Time	
	Net Differences				
	-Pre Birth	Post-Pre	Post-Birth	Partial Squared (ηp^2^)	*p*-Value
	Significant Difference; Eta^2^; (*p* Value)	Significant Difference; Eta^2^; (*p* Value)	Significant Difference; Eta^2^; (*p* Value)		
Emotional Attention	−0.49; 0.003; (0.66)	1.74; 0.02; (0.09)	−2.24; 0.06; (0.00)	0.024	0.21
Clarity of Feelings	3.11; 0.09; (0.01)	4.77; 0.14; (0.00)	1.66; 0.03; (0.13)	0.15	0.00
Emotional Repair	1.87; 0.04; (0.11)	2.70; 0.05; (0.06)	0.83; 0.00; (0.47)	0.05	0.07

Pre = period before childbirth or third trimester of pregnancy; Birth = first 24 h after birth; Post = Completion of the puerperium, first 8 weeks after childbirth; Eta^2^ = eta squared; *p* = Static significance (95%); TMMS = Trait-Goal Mood Scale.

## Data Availability

Data are contained within the article.
